# Review of the Effect of Surface Coating Modification on Magnesium Alloy Biocompatibility

**DOI:** 10.3390/ma15093291

**Published:** 2022-05-04

**Authors:** Xuan Guo, Yunpeng Hu, Kezhen Yuan, Yang Qiao

**Affiliations:** School of Mechanical Engineering, University of Jinan, Jinan 250022, China; guoxuan123125@163.com (X.G.); hhyypp82516@163.com (Y.H.); y2294_6050@163.com (K.Y.)

**Keywords:** magnesium alloy, biocompatibility, surface coating modification, corrosion resistance, implantable bio-metal materials

## Abstract

Magnesium alloy, as an absorbable and implantable biomaterial, has been greatly developed in the application field of biomaterials in recent years due to its excellent biocompatibility and biomechanics. However, due to the poor corrosion resistance of magnesium alloy in the physiological environment, the degradation rate will be unbalanced, which seriously affects the clinical use. There are two main ways to improve the corrosion resistance of magnesium alloy: one is by adding alloying elements, the other is by surface modification technology. Compared with adding alloy elements, the surface coating modification has the following advantages: (1) The surface coating modification is carried out without changing the matrix elements of magnesium alloy, avoiding the introduction of other elements; (2) The corrosion resistance of magnesium alloy can be improved by relatively simple physical, chemical, or electrochemical improvement. From the perspective of corrosion resistance and biocompatibility of biomedical magnesium alloy materials, this paper summarizes the application and characteristics of six different surface coating modifications in the biomedical magnesium alloy field, including chemical conversion method, micro-arc oxidation method, sol-gel method, electrophoretic deposition, hydrothermal method, and thermal spraying method. In the last section, it looks forward to the development prospect of surface coating modification and points out that preparing modified coatings on the implant surface combined with various modification post-treatment technologies is the main direction to improve biocompatibility and realize clinical functionalization.

## 1. Introduction

Medical metal materials play an important role in the repair and replacement of bone defects caused by bone joint diseases and trauma [1]. Magnesium is a macroelement needed in the human body, so the degradation of magnesium in the human body is generally considered as non-toxic [2]. At the same time, magnesium alloy can be dynamically degraded after being implanted into the human body, so excessive Mg^2^^+^ will be discharged out of the human body through the circulation system [3]. In addition, the elastic modulus of magnesium alloy implanted in the human body is similar to that of bone, so the stress shielding effect can be improved to a certain extent [4,5]. Compared with polymers, ceramics, and other medical implantable metals, magnesium alloy materials have good human biochemical compatibility, unique degradability, and reliable mechanical properties [6,7]. However, because the chemical properties of medical magnesium alloy are too active and the corrosion rate in the human body is too fast, the supporting strength of the material decreases sharply in a short time, and the biocompatibility is very poor, which affects its application effect [8]. Moreover, as a heterogeneous material, its safety needs to be further improved before it can be widely used in clinical orthopedics [9]. At present, research shows that the surface coating modification of magnesium alloy can not only improve the corrosion resistance of magnesium alloy but also improve its mechanical properties and biocompatibility, so it is of great significance to master the surface coating modification of different magnesium alloys [10,11,12]. In recent years, with the progress of research, the surface structure has been considered as one of the important factors affecting the combination of implants and bones [13,14], and its morphology can regulate the growth orientation of cells [15], and processing controllable surface structure is of great significance to improving the biocompatibility of materials [16]. Based on previous studies, this paper discusses the characteristics of different surface coating modification technologies of magnesium alloys and their effects on corrosion resistance and biocompatibility of magnesium alloys and discusses the future development trend of surface coating modification of biomedical magnesium alloys in the future.

## 2. Effect of Surface Coating Modification on Biocompatibility of Magnesium Alloy

Surface modification technology refers to the formation of protective coatings with different functions on the surface of substrate materials by various technological methods to achieve the desired purpose, which is a very important surface modification method in surface engineering technology [17]. A barrier can be formed by adding coating treatment on the metal surface to control the degradation rate of magnesium alloy [18]. The biocompatibility of magnesium alloy mainly means that magnesium alloy, as a bone implant, will not bring adverse reactions to the body on the basis of realizing connection, fixation, and maintaining normal healing of the body. In the whole healing process, magnesium alloy must first ensure that it can support the fracture site and ensure the normal healing of bones [19]. Second, the process of gradual corrosion and degradation of magnesium alloy in the human body should be at a moderate speed. Too fast will cause the bone not to heal in a short time, which will lead to treatment failure. Too slow will cause the patient’s treatment time to be too long [20], and the substances produced by corrosion and degradation should not be harmful to the human body. For example, hydrogen generated by the chemical reaction between magnesium electrochemical potential and electrolytes in an aqueous environment will lead to inflammation [21]. Finally, on the premise of ensuring basic function, magnesium alloy is treated on its surface to make it beneficial to cell growth, thus further improving biocompatibility. Common metal surface coating treatment methods include chemical conversion method, sol-gel method, micro-arc oxidation method, electrodeposition method, and hydrothermal treatment.

## 3. Effect of Chemical Transformation on Biocompatibility

Chemical conversion method is a method of converting the surface of the material itself into a coating, which belongs to the in-situ growth of the material and has good coating adhesion [22]. In the process of conversion coating, the substrate to be protected is immersed in the solution that reacts with the surface, changing the concentration of metal ions and the pH value of the interface of the metal solution so that the substrate material itself is converted into another material, forming a coating with good adhesion [23]. Chemical conversion method is a method with convenient production, small upfront investment, quick response, and controllable production conditions. Because the surface of the material itself is converted into a coating, the ceramic film has a strong bonding force, and it can transform one nano-material into another new nano-structure that is difficult to prepare directly, complex, and has unique properties. Therefore, this technology has a broad application prospect. Chemical conversion coating usually includes chromate coating, phosphate coating, fluorine-containing coating, etc. However, considering that chromate will leach out of the coating and lead to cancer, the coatings of chemical conversion method mainly focus on phosphate coating and fluorine-containing coating.

### 3.1. Study on Phosphate Coating

**Figure 1:**
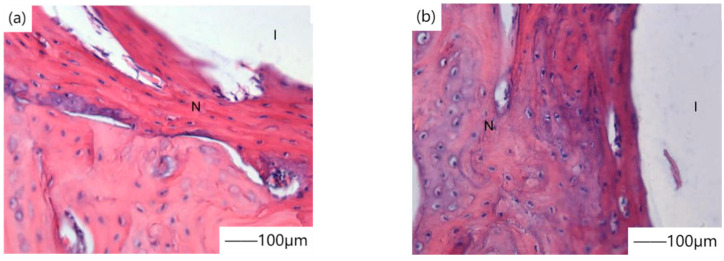
Comparison diagram of newly-born bone cells coated with (**a**,**b**) calcium phosphate around the culture. Reprinted with permission from Ref. [24]. Copyright 2019 Copyright Elsevier.

**Figure 2:**
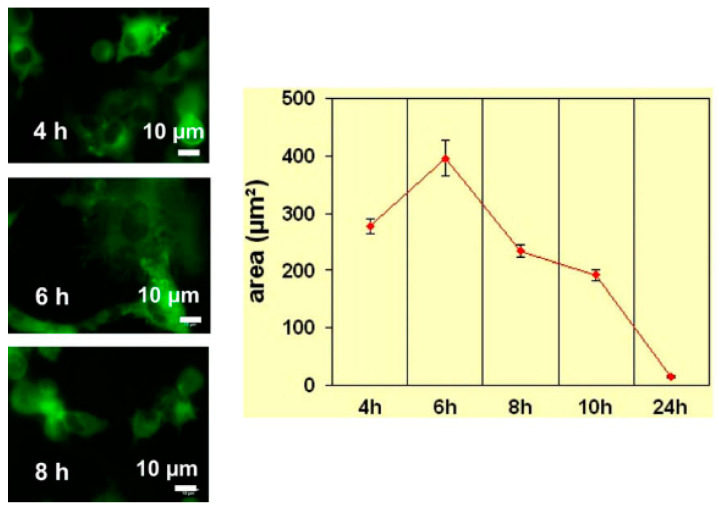
Mouse fibroblasts on Mg surface that had been pre-treated by soaking in M-SBF at 37 °C for 5 days. Fluorescence imaging of cells and cell spreading area as a function of culture time. Reprinted with permission from Ref. [25]. Copyright 2009 Copyright Elsevier.

In the study of biocompatibility of phosphate coating, the first thing to study is cell culture in vitro. Xu et al. [24] coated calcium phosphate coating on magnesium alloy surfaces by the chemical transformation method, and cell line L929 showed good growth rate and proliferation characteristics in in vitro cell experiments. Figure 1 shows the contrast diagram of new bone cells that were not coated with calcium phosphate coating after 4 weeks of culture. Calcium phosphate coated magnesium alloy was implanted in vivo to study the early bone reaction. The results showed that calcium phosphate coated magnesium alloy showed significantly improved bone conduction and osteogenesis ability in the first four weeks after operation. Calcium phosphate coated magnesium alloy provided significantly good surface bioactivity for magnesium alloy and promoted the early bone growth at the implant/bone interface. Lorenz et al. [25] studied the effect of calcium/magnesium phosphate coating on the surface of magnesium on the survival rate of mouse fibroblasts in cell culture experiments. After soaking in NaOH-SBF, a mixed calcium/magnesium phosphate coating was formed on pure magnesium. Compared with the magnesium surface simply soaked in NaOH solution, the cell survival rate was improved. Figure 2 shows the change of cell fluorescence imaging and cell diffusion area with culture time. The results show that chemical transformation with NaOH-SBF solution can improve the reactivity, chemical properties, and roughness of the material surface and provide a feasible strategy for cell survival on the magnesium surface. With the in-depth study of biocompatibility of magnesium alloy, people began to introduce implants into animals for research. Yang et al. [26] coated calcium phosphate coating on magnesium alloy (AZ31). The samples were implanted into rabbits to study the early bone reaction. This is because the slow biodegradation rate confirms the positive effect of phosphate coating.

To sum up, the reason why phosphate coating has good biocompatibility is that after the implant is implanted into the animal body, the surface begins to dissolve in body fluid and releases a large amount of calcium and phosphorus ions, which partially supersaturates the body fluid ions near the bone, which is beneficial to the formation and growth of bone cells. Furthermore, with the increase in time, the concentration of Mg^2+^ in body fluid increases, and it combines with calcium ions to form the nucleation of Ca-P compounds, which leads to the growth of Ca-P on magnesium alloy, which is beneficial for osteoblasts to adhere to the phosphate surface. Obviously, phosphate conversion coating has high temperature resistance, chemical stability, and excellent biocompatibility and is a good substitute for chromate as a biological coating. However, the phosphate conversion coating prepared on the surface of magnesium alloy by chemical conversion method generally has defects such as porosity and cracks, and the solution pollutes the environment, resulting in a high cost of treating the three wastes, which does not meet the needs of high-quality clean production and sustainable development strategy in China.

### 3.2. Study on Fluorine-Containing Coating

**Figure 3:**
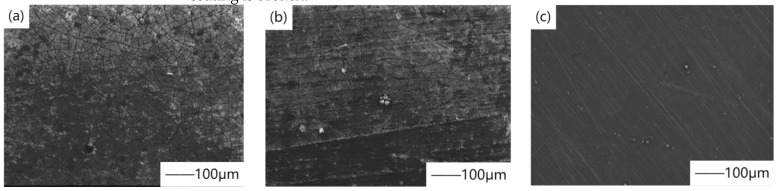
SEM surface morphology of the magnesium fluoride-coated materials after 11 days of immersion in DMEM: (**a**) PM Mg, (**b**) cast Mg, and (**c**) AZ31 alloy. Reprinted with permission from Ref. [27]. Copyright 2011 Copyright Elsevier.

In the research on fluorine-containing coating, the mechanism that magnesium fluoride coating can delay corrosion is mainly used. In the research on cell culture of magnesium alloy in vitro, Carboneras et al. [27] studied the biodegradation kinetics of powder metallurgy magnesium, cast magnesium, and magnesium alloy AZ31, which was evaluated by electrochemical impedance spectroscopy measurement in cell culture medium (DMEM). In order to reduce their degradation rate, chemical conversion treatment was carried out in hydrogen fluoride during the experiment to form magnesium fluoride coating. Figure 3 shows the SEM surface morphology of magnesium fluoride coating material after immersion in DMEM for 11 days. The results showed that magnesium fluoride coating slowed down the biodegradation rate, especially on cast magnesium and magnesium alloy AZ31, and delayed the corrosion time for at least one week. This is because the formed coating has inherent protective properties, such as high density, low water solubility, and high insulation caused by high impedance. Chiu et al. [28] used pure magnesium as the substrate and soaked it in hydrofluoric acid at room temperature to obtain a dense and crack-free magnesium fluoride coating with a thickness of 1.5μm on the surface. Magnesium fluoride coating is mainly composed of tetragonal magnesium difluoride, and the crystallite size is estimated to be several nanometers. The results show that with a good electrode impedance of 0.18 kΩcm^2^, the corrosion current density is reduced by 40 times. This is because the magnesium fluoride coating is chemically inert and can be used as an anticorrosive barrier coating. In a word, chemical conversion treatment of fluorinated coating is a simple and promising method to improve the corrosion resistance of magnesium in Hank’s solution, and it can also be used as the pretreatment step of subsequent coating. In the exploration of magnesium alloy as an implant in animals, Witte et al. [29] studied whether the extruded magnesium alloy LAE442 coated with magnesium fluoride was corroded and degraded by appropriate host reaction in rabbits. The results show that the fluorine-coated implant can effectively delay the corrosion of magnesium alloy and has an acceptable host reaction in rabbits. This is because the dense magnesium fluoride coating plays an isolation role, effectively reducing the hydrogen generated by the reaction between body fluid and magnesium alloy and reducing the toxic effect caused by hydrogen evolution. Moreover, the corrosion products formed in the coating materials are compounds rich in calcium and phosphorus, which are necessary for bone health, which also confirms the clinical application of magnesium fluoride coating as biodegradable implant. However, magnesium fluoride (MgF_2_) coating seems to stimulate local synovial tissue during the dissolution process, and pitting corrosion is larger than that of uncoated alloy after the coating is broken.

Fluoride conversion coating has good corrosion resistance, improves cell response and biocompatibility, and is an effective coating widely used in the biomedical field. However, although fluoride conversion coating can provide protection for magnesium alloy at the initial stage of implantation, it is difficult to provide long-term effective corrosion protection for magnesium alloy because the coating is very thin. At the same time, because excessive fluoride will have a negative impact on human bones, and the release process of fluoride ions during the degradation of magnesium implants and its toxicity to surrounding tissues are still unclear, the safety of fluoride coating still needs many clinical experiments to study.

## 4. Effect of Micro-Arc Oxidation on Biocompatibility

Micro-arc oxidation (MAO), also known as plasma electrolytic oxidation (PEO) or micro-plasma oxidation (MPO), is a high-pressure plasma-assisted anodizing process [30]. Micro-arc oxidation has the following advantages: (1) Improved surface hardness, with microhardness of 1000 to 2000 HV, up to 3000 HV, which is comparable to that of cemented carbide; (2) good wear resistance, which fundamentally overcomes the shortcomings of soft metal materials such as aluminum and magnesium alloy in application; (3) good insulation performance, with insulation resistance up to 100 mΩ; (4) the solution is environmentally friendly and meets the requirements of environmental protection discharge. Micro-arc oxidation electrolyte does not contain toxic substances and heavy metal elements, and it has strong anti-pollution ability and a high recycling rate, thus causing little environmental pollution; (5) the reaction is carried out at room temperature, which is convenient to operate and easy to master. In the coating surface modification process, the micro-arc oxidation process is often used for titanium alloy, and it plays an important role. In recent years, both of them have developed rapidly. The similarities are an alkaline oxidation environment, the conventional oxidation system includes phosphate electrolyte, and there is little difference in arc voltage and oxidation speed [31,32]. Moreover, after oxidation at 350–360 V for 15–20 min, the corrosion current of MAO coating on titanium alloy surface is lower than that of magnesium alloy, and the corrosion voltage is higher than that of magnesium alloy, so titanium alloy coating is more corrosion resistant [33,34], which also includes the application of titanium alloy. However, magnesium alloy is more suitable for bone plate because of its unique degradability, which can degrade in vivo to avoid the pain of secondary removal.

The morphology and chemical properties of MAO coating depend not only on the composition of electrolyte and properties of alloy, but also on processing parameters such as current density [35], voltage [36], heat treatment time [37], power supply mode, and loading parameters [38]. Generally, MAO coating on magnesium alloy consists of a porous outer layer and a thin barrier inner layer. Due to the discharge caused by the current breaking through the local growth layer in the process of micro-arc oxidation, characteristic micropores are produced on the surface of the coating. This porous coating provides certain corrosion protection for the magnesium substrate, and it can also act as an intermediate layer to improve the bonding force of the composite coating [39].

**Figure 4:**
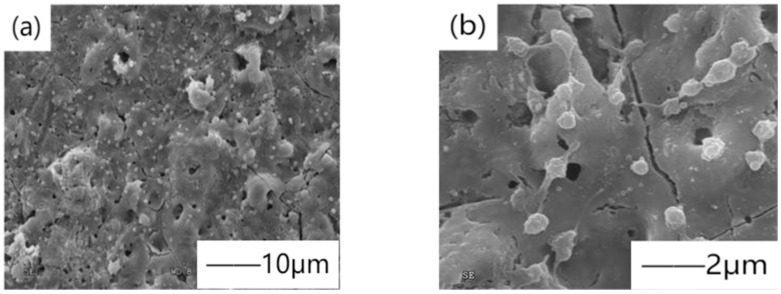
SEM images of blood platelet adhesion on the micro-arc oxidation (MAO) coating HA-containing coating: (**a**) SEM images with scale of 10μm, (**b**) SEM images with scale of 2μm. Reprinted with permission from Ref. [42]. Copyright 2018 Copyright Elsevier.

Yu et al. [40] formed ceramic bioactive coating on ZK61 magnesium alloy substrate by micro-arc oxidation, and the static corrosion test by soaking in simulated body fluid (SBF) verified that the coating could improve the corrosion resistance of the substrate. However, the static corrosion medium studied by Yu cannot effectively and truly approach the dynamic physiological flow state of body fluid in the human body. Han et al. [41] designed the flow field to simulate the biological corrosion performance of the human body in real physiological environment, providing a new scientific theoretical basis. However, the characteristic micropores formed by micro-arc oxidation also provide a path for corrosive solution, which leads corrosive ions to contact the magnesium matrix and react with it. The solutions are usually divided into two categories, one is to form self-sealing coating by adding elements or particles, and the other is to carry out post-treatment by a two-step method combined with other processes. Ding et al. [42] prepared a ceramic coating containing hydroxyapatite (HA) on biodegradable Mg_66_Zn_29_Ca_5_ magnesium alloy by micro-arc oxidation by adding hydroxyapatite (HA) particles in the electrolyte. Figure 4 shows the scanning electron microscope image of the micro-arc oxidation coating. The results showed that the biocompatibility was the best when HA concentration was 0.4 g/L. Li et al. [43] showed that by adding rare earth elements, the corrosion resistance is improved by reducing the active area of the substrate surface. With the deepening of research, in recent years, strontium [44], gallic acid [45], and copper [46], which can enhance bone formation and antibacterial effects, have been added to electrolyte solutions.

Post-treatment is carried out by a two-step method combined with other processes, one of which is chemical treatment such as the sol-gel method and electrophoretic deposition method, which will be summarized below. The other is physical treatment, such as sliding friction technology and ultrasonic cold forging. Hu et al. [47] combined sliding friction technology (SFT) developed by our research group with MAO, Yang et al. [48] combined with MAO by ultrasonic cold forging (UCFT), and Lavanya et al. [49] combined it with powder coating double layer, all of which improved the corrosion resistance of magnesium alloys.

The reason why micro-arc oxidation coating can delay corrosion is that magnesium alloy with MAO coating is immersed in Hanks, with the increase in soaking time, the thin barrier inner layer will prevent further penetration of the corrosion medium, resulting in slow corrosion rate. Moreover, in Hanks, the corrosion products obtained by spontaneous mineralization can realize the self-protection of the coating and slow down the corrosion rate of the coating. By improving the micro-arc oxidation process, adding HA particles or rare earth, strontium, and copper elements into the electrolyte can change the surface morphology, resulting in the increase in hydrophilicity, which reduces the absorption tendency of protein, platelets, and coagulation factors. High surface roughness provides favorable attachment points for cell growth and migration, thus improving the blood compatibility of implanted materials. Moreover, compared with ordinary MAO coating, the coating containing HA has fewer cracks and pores, which further inhibits the penetration of the corrosive medium and has good corrosion resistance [50,51]. The combination of sliding technology (SFT), ultrasonic cold forging (UCFT), and powder coating double layer with micro-arc oxidation is effective because of the grain refinement of the coating. The deformed layer with plastic deformation was obtained by SFT treatment, and the grain size of the uppermost layer of the deformed layer was refined to nano-scale, which could make the cells show an extended polygonal shape, have strong adhesion with the coating, and show many filamentary pseudopods. Through UCFT treatment, the surface of magnesium alloy can be nano-sized, the pore diameter can be reduced, the number of pores can be increased, the compactness can be improved, and the defects of film can be reduced.

Although micro-arc oxidation can control pores by adding polymer sealing coating and combining with other methods, the high experimental cost is still an important reason why this method is difficult to be widely applied. Moreover, the oxidation voltage is much higher than that of conventional anodic oxidation, so safety protection measures should be taken during operation. During the experiment, the temperature of electrolyte rises rapidly, and large-capacity refrigeration and heat exchange equipment is required, which requires a large investment in the early stage.

## 5. Effect of Sol-Gel Method on Biocompatibility

**Figure 5:**
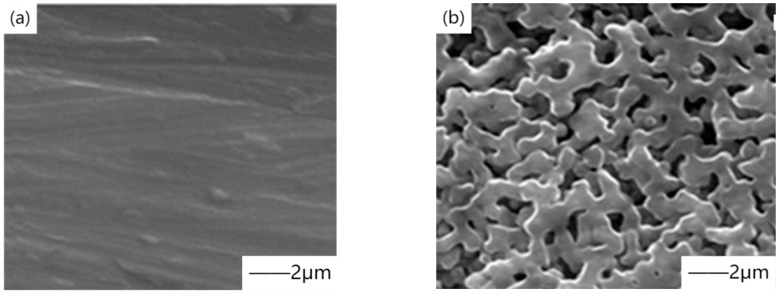
SEM analyses of (**a**) uncoated AZ31, (**b**) Nb2O5 coated AZ31 substrate. Reprinted with permission from Ref. [55]. Copyright 2014 Copyright Elsevier.

**Figure 6:**
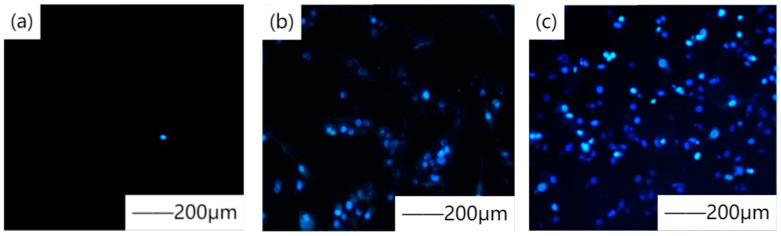
DAPI staining of nuclei of live osteoblast cells showing blue fluorescence on (**a**) uncoated and (**b**) Nb_2_O_5_ coated substrates after incubation with MG63 osteoblast cells for 24 h and (**c**) nuclei of live cells observed on Nb_2_O_5_ coated substrate after 48 h of incubation with MG63 osteoblast cells. (Ethidium bromide/acridine orange combined stain causes live cells to fluoresce green, whereas apoptotic cells cause the distinctive red-orange fluorescence.) Reprinted with permission from Ref. [55]. Copyright 2014 Copyright Elsevier.

Sol-gel method is a method in which liquid compounds with high chemical activity components undergo a series of chemical reactions to form gels and then oxidize into solids [52]. Sol-gel method is a general technology that has many advantages. (1) Because the raw materials are dispersed in the solvent, the reactants can be evenly mixed at the molecular level. When forming gel, it can be easily and evenly covered on the surface, especially suitable for irregular and complex surfaces. (2) Because of the step of solution reaction, it is easy to mix some trace elements uniformly and quantitatively, so as to achieve uniform doping at the molecular level. (3) Compared with the solid-phase reaction, the chemical reaction in solution is easier to carry out, and requires a lower synthesis temperature. It is generally believed that the diffusion of components in sol-gel system is in the nanometer range, whereas that in solid-phase reaction is in the micrometer range, so the purity of the formed coating is higher. Effective coatings with improved properties can be provided by changing the ratio of precursor to solvent, hydrolysis agent, curing temperature, hydrolysis rate, and deposition time [53]. There are two methods to prepare sol-gel: (a) inorganic and (b) organic. The inorganic method is to gel suspended colloidal particles with a particle size of 1–1000 nm to form a network. Nezamdoust et al. [54] used sol-gel coating containing different amounts of hydroxylated nano-diamond (HND) particles to treat magnesium alloy with corrosion resistance. Amaravathy et al. [55] synthesized niobium oxide (Nb_2_O_5_) coating on magnesium alloy by the sol-gel method. The surface characterization of the coating shows that the coating is composed of porous nanoparticles with a grain size of approximately 48 nm. Figure 5 shows the contrast of AZ31 with and without Nb_2_O_5_ coating. Niobium oxide coating has high microhardness and bonding strength, provides good surface anticorrosion protection, reduces degradation rate, and significantly enhances cell adhesion. Figure 6 shows the DAPI staining of osteoblast nuclei living on different magnesium alloy surfaces. Generally, the organic method is to dissolve metal monomer in organic solvent. Habib et al. [56] used tetraethoxysilane (TEOS) and methyltriethoxysilane (MTES) as raw materials to prepare sol-gel coating. The reason for the improvement of corrosion resistance is mainly the formation of a dense Si-O-Si network. Khramov et al. [57] prepared phosphate hybrid silane coating by mixing diethylphoxyethyl triethoxysilane and tetraethoxysilane in different molar ratios through hydrolysis and condensation. The enhancement of corrosion was due to the chemical reaction between phosphate and alloy surface, thus improving the hydrolysis stable P-O-Mg bond. In addition, the sol-gel method can not only combine with the matrix material at the molecular level to obtain uniform coating, but also be used as a sealing method for coatings with micropores and microcracks, such as MAO and CaP, to prepare mesoporous coatings [58,59]. Zhang et al. [60] prepared aminated hydroxyl cellulose (AHEC) coating on the surface after micro-arc oxidation by the sol-gel method, covering the pores of the micro-arc oxidation layer. Liu et al. [61] performed sol-gel post-treatment on the CaP coating to repair the pore gap of the coating. 

The inorganic method can alleviate the corrosion because the hydroxide formed on the surface of the coating enhances the surface energy and improves the hydrophilicity through mechanical interlocking with ions. The nano-scale roughness of the coating is conducive to cell adhesion, porosity is conducive to bone integration, provides more contact surfaces for cell pseudopodia, and provides a way for cell nutrition penetration, so the morphology, proliferation rate, and expansion of cells are improved. The reason why the organic method can alleviate corrosion is that the formed silane-based sol-gel coating has low electrical reaction sensitivity with Mg, is easy to be chemically modified, and its chemical properties have low cytotoxicity to cells. Silicon and oxygen elements form a dense Si-O-Si network, which has stronger adhesion. In addition, the sealing moon, as a microporous and microcrack coating, can delay corrosion, because the permeable water of the sol condensation solvent diffuses, and the coating is preferentially attacked in the presence of water. At the same time, hydrogen generated by corrosion can be discharged through pores of the coating, and the hydrophilic porous structure on the surface promotes cell proliferation. Therefore, as a porous organic material, sol coagulation can not only act as a sealing layer, but can also provide good conditions for cell adhesion and proliferation [62]. However, the whole sol-gel process takes a long time, often several days or weeks. There are a lot of micropores in the gel, which will escape many gases and organic substances and cause shrinkage. The internal stress generated in the heat treatment process often leads to the cracking of the coating and reduces the corrosion resistance of the coating. Therefore, sol-gel coating technology needs further optimization, and how to control the coating thickness by changing parameters in the future to protect magnesium alloy matrix is a big development direction.

## 6. Effect of Electrophoretic Deposition on Biocompatibility

**Figure 7:**
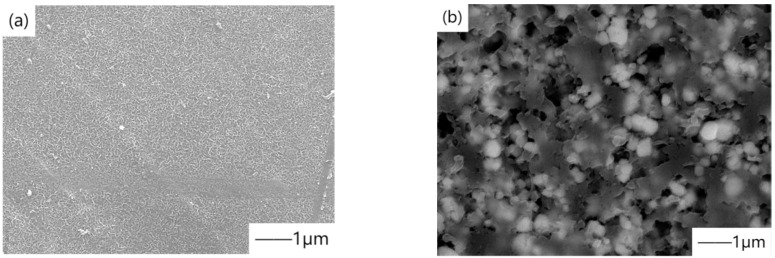
Comparison of SEM micrographs of Mg alloy coated with HAP and uncoated with HAP: (**a**) Mg alloy uncoated with HAP, (**b**) Mg alloy coated with HAP. Reprinted with permission from Ref. [65]. Copyright 2020 Copyright Elsevier.

Electrophoretic deposition (EPD) is a process in which particles in suspension are deposited on the surface of a substrate using a DC electric field. The specific operation process generally means that under the action of the external DC electric field, the particles in the suspension move towards one end with opposite charges and gradually deposit a coating on the substrate material. The most critical factor affecting the surface morphology of the coating is suspension, including particle size, liquid dielectric constant, conductivity, medium viscosity, electromotive force, and stability of suspension [63]. In addition, deposition time, applied voltage, solid concentration in suspension, and substrate conductivity also have significant influence on the electrophoresis process [64]. Electrophoretic deposition has the advantage of forming a layer in a short time, requiring only simple equipment. In the application of electrophoretic deposition, hydroxyapatite coating is typically prepared. Antoniac et al. [65] prepared a uniform and continuous HAP coating with a thickness of approximately 15–16μm on the surface of Mg-Zn-Mn biodegradable alloys (ZMX410 and ZM21) by controlling the electrophoretic deposition parameters and compared the biodegradation rates before and after coating. Figure 7 shows the SEM micrographs of Mg alloy without coating and coated with HAP. The results show that the coating reduces the corrosion current density and improves the biocompatibility. This is because carbonation leads to the improvement of surface hydrophilicity and polarization resistance, which effectively improve the corrosion resistance of Mg-Zn-Mn alloy in a simulated environment. In addition, Wang et al. [66] prepared an HA layer with biological activity on MAO coating by the EPD method. MAO coating surface is loose and porous, which provides a good position for the deposition of HA particles, and HA particles will not easily fall off the sample surface, thus improving the corrosion resistance.

Electrophoretic deposition has many advantages: (1) The preparation of HA coating is carried out in a mild environment, so there is no thermal stress at the interface between the substrate and the coating. This process can avoid the phase change and brittle fracture caused by high-temperature spraying, which is conducive to enhancing the bonding strength between the substrate and the coating. (2) Electrophoretic deposition is a non-linear process, so it is not limited by the shape and uniformity of the magnesium alloy surface. Ceramic coating can be prepared on the substrate with complex shape and porous surface, and the coating is plump, uniform, flat, and smooth. Electrophoresis has high dispersion ability, and even in the concave part of the product, a completely uniform protective film can be formed. (3) The thickness of the coating can vary in a large range. By accurately controlling the thickness and shape of the coating, it can vary from one micron to one hundred microns. The thickness of the coating can be controlled by adjusting different operating voltages to achieve extremely high corrosion resistance. (4) The deposition is fast, the yield is high, and it only takes a few seconds or minutes. The changeable preparation technology makes it have a broad commercial value. However, during electrophoretic deposition, HA particles are unevenly dispersed, and it is easy to form hydrogen bubbles at the magnesium surface, which seriously affects coating density and interfacial bonding strength. At the initial stage of electrophoresis, the investment is relatively large, and the workpiece that can be made can only be conductive. Of course, it can also be electrophoresed on plastic now, but a conductive layer has to be added. In the future, coatings with better biocompatibility and cell compatibility can be developed by combining with other methods to achieve better application in organisms.

## 7. Effect of Hydrothermal Method on Biocompatibility

Hydrothermal method refers to a method of preparing materials by dissolving and recrystallizing powder with water as solvent in a completely sealed pressure vessel. The main difference between the hydrothermal method and other wet chemical methods, such as the sol-gel method, lies in temperature and pressure. Hydrothermal methods usually use a temperature between 130 °C and 250 °C, and the corresponding water vapor pressure is 0.3 ~ 4 MPa. The coating prepared by hydrothermal method has the following advantages: (1) The coating can be directly obtained without high-temperature calcination, which avoids grain growth, defect formation, and impurity introduction during calcination, so the prepared coating has high sintering activity. (2) The prepared powder has the advantages of complete grain development, small particle size, uniform distribution, and light particle agglomeration, and it can be used as cheaper raw materials. The crystal structure, crystal morphology, and grain purity of nanoparticles can be controlled by adjusting the reaction conditions. The particle size range of the obtained powder materials is usually 0.1 μm to several microns, and some of them can reach tens of nanometers. Uniform coating can be prepared on surfaces with nonlinear and complex shapes. (3) The prepared calcium phosphate coating has high interfacial bonding strength and density, which is conducive to significantly improving the corrosion resistance of magnesium alloy biomaterials.

**Figure 8:**
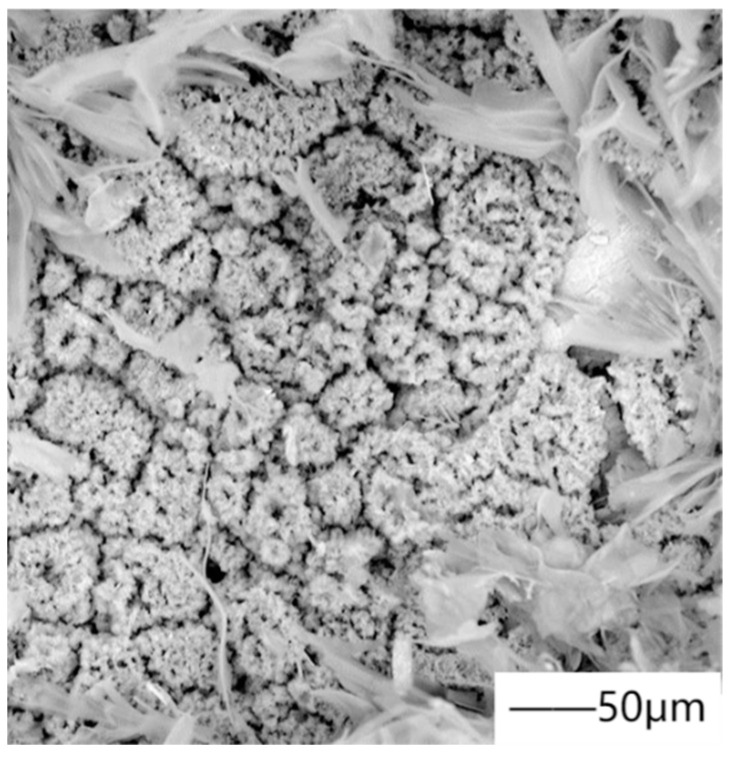
Shows the SEM micrograph of superhydrophobic hydroxyapatite coating. Reprinted with permission from Ref. [67]. Copyright 2018 Copyright Elsevier.

Compared with other technologies, the principle of preparing calcium phosphate (Ca-P) coating, especially hydroxyapatite coating (HAP), on the surface of magnesium alloy by hydrothermal treatment is relatively simple, and the operational device is easy to control. Therefore, more and more scholars use this method to carry out surface modification treatment on implantable biomedical magnesium alloy. Kang et al. [67] deposited superhydrophobic hydroxyapatite coating on magnesium alloy (Mg-Gd-1.5Nd-0.3Zn-0.3Zr) containing stearic acid and calcium phosphate compound by the one-step hydrothermal method. Figure 8 shows the SEM micrograph of superhydrophobic hydroxyapatite coating, and the test results show that the coating has low corrosion current density. This is because the superhydrophobic coating can make air stay in the layered structure of the coating, effectively reduce the contact between corrosive solution and the surface, and inhibit the exchange between corrosive ions and the surface. With the deepening of research, there have been many modified HAP in recent years. Zhou et al. [68] prepared a new type of hydroxyapatite coating which is denser than ordinary HA coating through the induction of poly dopamine, making it more corrosion-resistant. Peng et al. [69] enhanced the osteoinductivity of HA coating by adding zinc oxide nanoparticles with antibacterial activity. Yang et al. [70] introduced cationic surfactant tetradecyl trimethyl ammonium bromide (TTAB) for the first time and in-situ hydrothermally synthesized dense anticorrosive magnesium–aluminum layered double hydroxide films on AZ31 magnesium alloy. In addition, hydrothermal method is often combined with chemical conversion method [71], micro-arc oxidation method [72], and sol coagulation method [73] to improve the coating performance.

The core of the hydrothermal method is to select biocompatible materials with functional groups that coordinate with calcium ions, such as polydopamine, zinc oxide nanoparticles, amino acids, etc., which can chelate with calcium ions. The preparation of superhydrophobic coating can effectively improve the corrosion resistance of magnesium alloy, but the effect of superhydrophobic coating on the biological properties of magnesium alloy needs further study. In addition, the composite coating formed by other methods can also effectively improve the antibacterial and bone-guiding properties of calcium phosphate coatings, synthesize calcium phosphate coatings with different morphologies and phase compositions, significantly refine the grains of artificially synthesized HA crystals, and reduce their clustering effect [74]. It is worth noting that in order to protect the magnesium alloy coating for a long time, the density, thickness, solubility, and antibacterial ability of the coating must be comprehensively considered. These abilities of coatings are closely related to their chemical composition and micro-morphology, which directly affect the degradation rate. Therefore, it is a future development trend to design HA coating with comprehensive ability and strong bonding strength between coating and Mg matrix. However, the disadvantages of the hydrothermal method are obvious. The process is carried out in a closed container, and the growth process, which is not intuitive, cannot be observed. Moreover, it requires high equipment requirements, has great technical difficulty (strict control of temperature and pressure), and requires high-temperature and high-pressure steps, which makes it more dependent on production equipment. Therefore, the hydrothermal method tends to develop at low-temperature and low-pressure, that is, the temperature is lower than 100 °C and the pressure is close to one standard atmospheric pressure. Generally, only oxide powders can be prepared by the hydrothermal method, but there is a lack of in-depth research on the control of influencing factors of crystal nucleation and crystal growth, and no satisfactory conclusion has been reached.

## 8. Effect of Thermal Spraying Method

Thermal spraying is the use of high temperature and high speed to melt powder or metal wires as raw materials and deposit one material on the surface of another [75]. According to different heat sources, thermal spraying can be divided into flame thermal spraying, arc thermal spraying, plasma spraying, etc. Among them, plasma spraying (PSP) is the most common one in magnesium alloy surface modification. Plasma spraying systems consist of electric control power supply, plc-based operator control station, gas mass flow system, closed-loop water cooling system, powder feeder, and plasma torch. The unique features of plasma spraying process are: (1) it can melt a variety of metals, ceramics, or composite materials [76,77]; (2) the deposition rate is high [78]; (3) the obtained coating is uniform [79]; and (4) the coating can be controlled by a variety of parameter settings [80]. Most importantly, the particle velocity of plasma spraying is higher than that of flame spraying and arc spraying, so the coating is denser and the surface morphology is finer [81].

**Figure 9:**
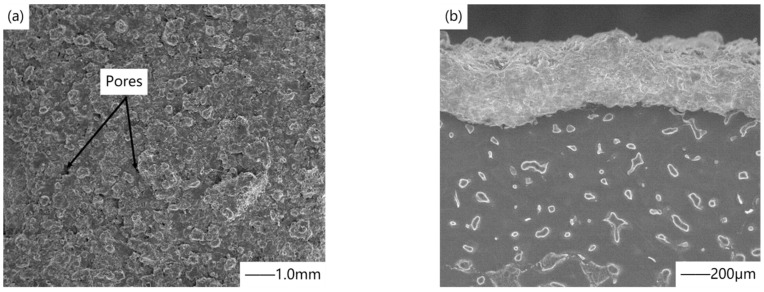
(**a**) Surface morphology of coating, (**b**) microstructure of coating section. Reprinted with permission from Ref. [82]. Copyright 2018 Copyright Elsevier.

In the preparation of medical magnesium alloy coating, spraying HA particles is the most common. Gao et al. [82] improve the corrosion resistance and bioactivity of magnesium alloy by plasma spraying hydroxyapatite coating. Figure 9 shows the surface morphology and cross-section microstructure of the coating. In recent years, with the deepening of research, everyone began to improve HA coating. Yao et al. [83] added zinc to hydroxyapatite. The corrosion rate of zinc was lower than that of magnesium, and zinc had a positive effect on bone mineralization and cell protein synthesis, thus improving the long-term viability and bone binding ability of the HA coating. Singh et al. [84] successfully sprayed Ta/Ti coating on AZ31B magnesium alloy by the plasma spraying process, which has high hardness and reduced wear rate. This behavior is attributed to the high passivation property of cold sprayed Ta/Ti coatings and the rough surface (island shape) of these coatings, which can provide proper nucleation sites for the formation and growth of calcium phosphate compounds in Hanks solution, and the dense structure of Ta layer hinders the penetration of corrosive solution. Mohajernia et al. [85] prepared a hydroxyapatite coating containing multi-walled carbon nanotubes (MWCNTs). Due to the high melting point of MWCNTs, they remained intact during plasma spraying and acted as a bridge between melted/semi-melted fragments, thus improving the fracture toughness of the HAP coating.

Plasma spraying is mainly used in medical magnesium alloy to enhance its surface hardness. The grain size grows, the strengthening phase disappears, and the grain boundary strengthening phase grows, which is the fundamental reason for the hardness enhancement of the alloy. Moreover, the plasma spraying speed is very high, so the coating is denser and the surface morphology is finer. Specifically, during plasma spraying, molten HA particles hit magnesium alloy and rapidly solidified to form an amorphous phase, partially decomposed to form β-Ca_3_(PO_4_)_2_, and the rest of the HA particles cooled to form HA phase, so the coating consists of amorphous phase, β-Ca_3_(PO_4_)_2_, and HA phase. Compared with HA, amorphous phase and β- Ca_3_(PO_4_)_2_ have higher solubility and degradation rate. The generated calcium and phosphorus ions react with protein molecules of bone cells, stimulating the growth of bone, and making implant materials form chemical-biological combination with bone, but at the same time, it also leads to the instability of the coating under the combined action of load and corrosion. The HA crystal is composed of unmelted or partially melted raw materials and recrystallized molten particles, which have a slow cooling rate, so they are not transformed into the amorphous phase. The dissolution rate of crystals in vivo and in vitro is slow, which is not conducive to bone growth, but it improves the mechanical properties of the coating and ensures the lasting bonding strength, which is particularly important at the interface between the coating and the implant. From the surface morphology of the coating, it can be seen from Figure 9 that the coating is composed of flat particles and a small amount of pores. The pores and rough structure of the plasma sprayed coating not only change the mechanical transmission mode of bone/implant, but also facilitate the climbing, growth, migration, and other activities of bone cells. For the development of HA coating in recent years, the modification is mainly aimed at improving the compactness of coating and reducing pore cracks. By adding Zn into HA coating by plasma spraying technology, ZnO clusters are formed on the surface of the coating, which enhances the bonding between particles, and the dense double-layer structure formed hinders the penetration corrosion of electrolyte to magnesium alloy substrate. According to the difference of thermal conductivity between HA and Ta, adding Ta can adjust the crystallinity of the coating, make the surface relatively flat, reduce the fused solidified particles, and improve the protective ability of the surface. Multi-walled carbon nanotubes (MWNTs) can improve the fracture toughness of the coating and limit the crack propagation, because they act as a bridge, and more energy is required to destroy it. Moreover, the existence of MWNTs will reduce the content of the secondary phase, thus affecting the fracture mechanism. However, the HA coating formed by plasma spraying has rough micro-scale, pores, and cracks, and poor bone conductivity. The high temperature in the processing leads to the decomposition of the calcium phosphate phase and the formation of the amorphous phase, and the purity and crystallinity of the coating are low, which accelerates the dissolution of the HA coating, leading to its loss of synchronization with the life of the implant. As far as the development of plasma spraying metal-based ceramic coatings is concerned, in order to further improve the density of coatings and reduce the cost of plasma spraying ceramic coatings, efforts should be made in the following aspects. First, optimize the coating composition and process parameters, and combine with more methods. Second, according to the requirements of different parts, a new type of environmentally friendly hole sealing agent was developed. Finally, while meeting different requirements, the production equipment is optimized and the production cost is reduced. Among many preparation methods of nano-ceramic coatings, thermal spraying technology is the most likely to produce market benefits in a short time.

## 9. Summary and Prospect

In this paper, six surface modification methods—chemical conversion method, micro-arc oxidation method, sol-gel method, electrophoretic deposition, hydrothermal method, and thermal spraying method—are reviewed. Chemical conversion method has good binding force because its own material is converted into coating. Micro-arc oxidation method combines with sol-gel method, which is often used as sealing layer, to obtain better coating. Electrophoretic deposition is not limited by the shape of magnesium alloy and can quickly form uniform and smooth coating. Because the hydrothermal method does not need high-temperature calcination treatment, the coating has high sintering activity, and thermal spraying improves metallographic structure and corrosion resistance by melting various materials. To compare the differences of the six methods more conveniently, their definitions, advantages and disadvantages, characteristics, and the number of literature publications are summarized in Table 1. Because magnesium alloy is implanted into the body as a bone plate, it must have strong mechanical properties, good corrosion resistance, and biocompatibility. At present, the most studied method is coating priming by micro-arc oxidation and then post-treatment by sol-gel method. However, the cost of this method is much higher than that of hydrothermal method and thermal spraying method. Considering that the coating of the implant should not fall off and be uniform, chemical conversion method and electrophoretic deposition can solve this problem well. In order to understand the development of the six methods, the number of publications was searched in the Web of Science database. There were 992 articles on the chemical conversion method, and the most published in 2019 was about 120. Micro-arc oxidation method (693) published the most in 2019, about 80 articles. Sol method (559) published the most in 2018, about 60 articles. Electrophoretic deposition method (618) published the most in 2019, about 80 articles. Hydrothermal method (394) published the most in 2021, with about 65 articles. Thermal spraying method (188) published the most in 2019, about 30 articles. However, these technical methods are not very mature in three aspects. (1) Biological safety: At present, most of the research on medical coated magnesium alloy is in vitro research, and only a few alloys are implanted into animals. However, the actual internal environment of the human body is more complicated, and it is impossible to accurately grasp the problems such as excessive hydrogen evolution, inflammation, and degradation of metal elements poisoning, and there is still a long way to go before clinical application. (2) Surface properties: Medical coated magnesium alloy has different functions due to different implantation sites. For example, the surface morphology of magnesium alloy bone plate needs to be conducive to the growth of cells, and the surface of magnesium alloy vascular support tube needs antithrombotic performance, etc. At present, although there are many methods to form coatings, it is difficult for the morphology formed on the surface to reach the ideal state. (3) Mechanical properties: At present, the research of medical coated magnesium alloy is mainly used as bone screws, but a single coating cannot provide stable degradation protection and cannot achieve the purpose of effectively connecting broken bones.

In view of the above problems, the development trend of medical coated magnesium alloy in the future is as follows: (1) Mastering the degradation mechanism of medical coated magnesium alloy through in vitro research and animal in vivo research, and studying the degradation conditions in human body through corrosion simulation, the coating can avoid using metal elements that may lead to poisoning, and can alleviate the precipitation of hydrogen. Coating magnesium alloy for medical use will reduce the cost on the basis of green production, and it will be widely used in clinical settings. (2) To improve the pertinence of magnesium alloy coating, various methods can be used at the same time to form ideal coating with pore size, surface roughness, hydrophilicity, and hydrophobicity. (3) Through the simulation of corrosion mechanics, the stress situation of each part is judged, and the mechanical properties (such as hardness, strength, etc.) are enhanced by multi-coating superposition in places with large stress. Multi-coating superposition is the development trend in the future, which makes medical coated magnesium alloy widely used for flexible coating. In a word, surface coating modification has created the possibility for further application of magnesium alloy in the medical field. It is believed that with the continuous development of surface coating modification technology of magnesium alloy, magnesium alloy will play a greater role in the field of biomedical valuable materials.

## Author Contributions

Conceptualization, Y.Q. and X.G.; methodology, X.G.; software, Y.H.; validation, Y.Q., Y.H. and K.Y.; formal analysis, K.Y.; investigation, K.Y.; resources, Y.Q.; data curation, Y.H.; writing—original draft preparation, X.G.; writing—review and editing, X.G.; visualization, K.Y.; supervision, K.Y.; project administration, Y.H.; funding acquisition, Y.H. All authors have read and agreed to the published version of the manuscript.

## Figures and Tables

**Table 1:** Comparison of modification methods of medical magnesium alloy surface coating.

Method	Chemical Conversion	Microarc Oxidation	Sol Coagulation	Electrophoretic Deposition	Hydrothermal Method	Thermal Spraying
Concept	The material itself is transformed into a coating through chemical changes.	High-voltage plasma assisted anodic oxidation process.	The liquid is converted into gel and then oxidized into a solid.	Particles are deposited on the surface of the substrate by direct current electric field.	With water as solvent, the powder is dissolved and recrystallized.	The powder is deposited on the surface at high temperature and high speed.
Adv.	The coating has good binding force and low cost.	With high hardness, it is often used as the base coat of the first step.	Uniform film formation, often used as the second step sealing coating.	Uniform coat can be prepared on a substrate with complex shape.	Avoids impurity entry and defect growth in that calcination process.	High deposition rate, uniform coating.
Disad.	Porous, cracked, and pollutes the environment.	Characteristic micropores make it corrosion-resistant.	Results in thermal stress cracking of the coating.	Large investment, difficult process control.	High equipment requirements and technical difficulties.	Poor bonding strength and environmental pollution.
Feature	Self-transformation, good binding force.	Coating with high hardness and abrasion resistance.	As the second step of sealing coating.	Avoid embrittlement caused by high temperature.	Coating has high sintering activity.	Improving metallographic structure and corrosion resistance.
Number	992	693	559	618	394	188

## Data Availability

Not applicable.

## References

[1] Farraro F.K., Kim E.K., Woo L.S., Flowers R.J., McCullough B.M. (2014). Revolutionizing orthopaedic biomaterials: The potential of biodegradable and bioresorbable magnesium-based materials for functional tissue engineering. J. Biomech..

[2] Alaneme K.K., Okotete A.E. (2017). Enhancing plastic deformability of Mg and its alloys—A review of traditional and nascent developments. J. Magnes. Alloy..

[3] Atrens A., Song G.L., Cao F., Shi Z., Bowen P.K. (2013). Advances in Mg corrosion and research suggestions. J. Magnes. Alloy..

[4] Moghaddam N.S., Andani M.T., Amerinatanzi A., Haberland C., Huff S., Miller M., Dean D. (2016). Metals for bone implants: Safety, design, and efficacy. Biomanuf. Rev..

[5] Sumner D.R. (2015). Long-term implant fixation and stress-shielding in total hip replacement. J. Biomech..

[6] Atrens A., Johnston S., Shi Z., Dargusch M.S. (2018). Understanding Mg corrosion in the body for biodegradable medical implants. Scr. Mater..

[7] Hermawan H., Dubé D., Mantovani D. (2010). Degradable metallic biomaterials: Design and development of Fe-Mn alloys for stents. J. Biomed. Mater. Res. Part A.

[8] Bairagi D., Mandal S. (2021). A comprehensive review on biocompatible Mg-based alloys as temporary orthopaedic implants: Current status, challenges, and future prospects. J. Magnes. Alloy..

[9] Razavi M., Huang Y. (2019). Assessment of magnesium-based biomaterials: From bench to clinic. Biomater. Sci..

[10] Surmeneva M.A., Vladescu A., Cotrut C.M., Tyurin A., Pirozhkova T., Shuvarin I., Elkin B., Oehr C., Surmenev R. (2018). Effect of parylene C coating on the antibiocorrosive and mechanical properties of different magnesium alloys. Appl. Surf. Sci..

[11] Elkamel R.S., Fekry A.M., Ghoneim A.A., Filippov L.O. (2022). Electrochemical Corrosion Behaviour of AZ91E Magnesium Alloy by means of Various Nanocoatings in Aqueous Peritoneal Solution: In Vitro and In Vivo Studies. J. Mater. Res. Technol..

[12] El-Kamel R.S., Ghoneim A.A., Fekry A.M. (2019). Electrochemical, biodegradation and cytotoxicity of graphene oxide nanoparticles/polythreonine as a novel nano-coating on AZ91E Mg alloy staple in gastrectomy surgery. Mater. Sci. Eng. C.

[13] Prabhu D.B., Gopalakrishnan P., Ravi K.R. (2020). Morphological studies on the development of chemical conversion coating on surface of Mg-4Zn alloy and its corrosion and bio mineralisation behaviour in simulated body fluid. J. Alloys Compd..

[14] Devgan S., Sidhu S.S. (2019). Evolution of surface modification trends in bone related biomaterials: A review. Mater. Chem. Phys..

[15] Li J.A., Chen L., Zhang X.Q., Guan S.K. (2020). Enhancing biocompatibility and corrosion resistance of biodegradable Mg-Zn-Y-Nd alloy by preparing PDA/HA coating for potential application of cardiovascular biomaterials. Mater. Sci. Eng. C.

[16] Huynh V., Ngo N.K., Golden T.D. (2019). Surface activation and pretreatments for biocompatible metals and alloys used in biomedical applications. Int. J. Biomater..

[17] Surmeneva M.A., Ivanova A.A., Tian Q., Pittman R., Jiang W., Lin J., Liu H.H., Surmenev R.A. (2019). Bone marrow derived mesenchymal stem cell response to the RF magnetron sputter deposited hydroxyapatite coating on AZ91 magnesium alloy. Mater. Chem. Phys..

[18] Surmeneva M.A., Mukhametkaliyev T.M., Khakbaz H., Surmenev R.A., Kannan M.B. (2015). Ultrathin film coating of hydroxyapatite (HA) on a magnesium–calcium alloy using RF magnetron sputtering for bioimplant applications. Mater. Lett..

[19] Tian L., Tang N., Ngai T., Wu C., Ruan Y., Huang L., Qin L. (2019). Hybrid fracture fixation systems developed for orthopaedic applications: A general review. J. Orthop. Transl..

[20] Chandra G., Pandey A. (2020). Biodegradable bone implants in orthopedic applications: A review. Biocybern. Biomed. Eng..

[21] Wan P., Tan L., Yang K. (2016). Surface modification on biodegradable magnesium alloys as orthopedic implant materials to improve the bio-adaptability: A review. J. Mater. Sci. Technol..

[22] Hornberger H., Virtanen S., Boccaccini A.R. (2012). Biomedical coatings on magnesium alloys-A review. Acta Biomater..

[23] Chen X.B., Birbilis N., Abbott T.B. (2011). Review of Corrosion-Resistant Conversion Coatings for Magnesium and Its Alloys. Corrosion.

[24] Xu L.P., Pan F., Yu G., Yang L., Zhang E., Yang K. (2009). In Vitro and In Vivo evaluation of the surface bioactivity of a calcium phosphate coated magnesium alloy. Biomaterials.

[25] Carla L., Johannes G.B., Philip K. (2009). Effect of surface pre-treatments on biocompatibility of magnesium. Acta Biomater..

[26] Yang J.X., Cui F.Z., Lee I.S., Zhang Y., Yin Q., Xia H., Yang S. (2012). In Vivo biocompatibility and degradation behavior of Mg alloy coated by calcium phosphate in a rabbit model. J. Biomater. Appl..

[27] Carboneras M., Garcia-Alonso M.C., Escudero M.L. (2011). Biodegradation kinetics of modified magnesium-based materials in cell culture medium. Corros. Sci..

[28] Chiu K.Y., Wong M.H., Cheng F.T. (2007). Characterization and corrosion studies of fluoride conversion coating on degradable Mg implants. Surf. Coat. Technol..

[29] Witte F., Fischer I.J., Nellesen J., Vogt C., Vogt J., Donath T., Beckmann F. (2010). In Vivo corrosion and corrosion protection of magnesium alloy LAE442. Acta Biomater..

[30] Zhu Y., Gao W., Huang H., Chang W., Zhang S., Zhang R., Zhang Y. (2019). Investigation of corrosion resistance and formation mechanism of calcium-containing coatings on AZ31B magnesium alloy. Appl. Surf. Sci..

[31] Shin K.R., Ko Y.G., Shin D.H. (2012). Surface characteristics of ZrO_2_-containing oxide layer in titanium by plasma electrolytic oxidation in K_4_P_2_O_7_ electrolyte. J. Alloys Compd..

[32] Yigit O., Ozdemir N., Dikici B., Kaseem M. (2021). Surface properties of graphene functionalized TiO_2_/nHA hybrid coatings made on Ti6Al7Nb alloys via plasma electrolytic oxidation (PEO). Molecules.

[33] Zehra T., Kaseem M., Hossain S., Ko Y.G. (2021). Fabrication of a protective hybrid coating composed of TiO_2_, MoO_2_, and SiO_2_ by plasma electrolytic oxidation of titanium. Metals.

[34] Lu J.P., Cao G.P., Quan G.F., Wang C., Zhuang J.J., Song R.G. (2018). Effects of voltage on microstructure and corrosion resistance of micro-arc oxidation ceramic coatings formed on KBM10 magnesium alloy. J. Mater. Eng. Perform..

[35] Ezhilselvi V., Nithin J., Balaraju J.N., Subramanian S. (2016). The influence of current density on the morphology and corrosion properties of MAO coatings on AZ31B magnesium alloy. Surf. Coat. Technol..

[36] Yu H., Dong Q., Dou J., Pan Y., Chen C. (2016). Preparation of Si-containing oxide coating and biomimetic apatite induction on magnesium alloy. Appl. Surf. Sci..

[37] Yong J., Li H., Li Z., Chen Y., Wang Y., Geng J. (2021). Effect of (NH_4_)_2_ZrF_6_, voltage and treating time on corrosion resistance of micro-arc oxidation coatings applied on ZK61M magnesium alloys. Materials.

[38] Yao J.T., Wang S., Zhou Y., Dong H. (2020). Effects of the power supply mode and loading parameters on the characteristics of micro-arc oxidation coatings on magnesium alloy. Metals.

[39] Xiong Y., Yang Z., Hu X., Song R. (2019). Bioceramic coating produced on AZ80 magnesium alloy by one-step microarc oxidation process. J. Mater. Eng. Perform..

[40] Yu H., Dong Q., Dou J., Pan Y., Chen C. (2016). Structure and in vitro bioactivity of ceramic coatings on magnesium alloys by microarc oxidation. Appl. Surf. Sci..

[41] Han L., Li X., Xue F., Chu C., Bai J. (2019). Biocorrosion behavior of micro-arc-oxidized AZ31 magnesium alloy in different simulated dynamic physiological environments. Surf. Coat. Technol..

[42] Ding H.Y., Li H., Wang G.Q., Liu T., Zhou G.H. (2018). Bio-Corrosion Behavior of Ceramic Coatings Containing Hydroxyapatite on Mg-Zn-Ca Magnesium Alloy. Appl. Sci..

[43] Jianzhong L., Yanwen T., Zuoxing G.U.I., Huang Z. (2008). Effects of rare earths on the microarc oxidation of a magnesium alloy. Rare Met..

[44] Sedelnikova M.B., Sharkeev Y.P., Tolkacheva T.V., Khimich M.A., Bakina O.V., Fomenko A.N., Epple M. (2020). Comparative Study of the structure, properties, and corrosion behavior of Sr-containing biocoatings on Mg0.8Ca. Materials.

[45] Lee H.P., Lin D.J., Yeh M.L. (2017). Phenolic modified ceramic coating on biodegradable Mg alloy: The improved corrosion resistance and osteoblast-like cell activity. Materials.

[46] Ahmed M., Qi Y., Zhang L., Yang Y., Abas A., Liang J., Cao B. (2020). Influence of Cu^2+^ ions on the corrosion resistance of AZ31 magnesium alloy with microarc oxidation. Materials.

[47] Huo W., Lin X., Lv L., Cao H., Yu S., Yu Z., Zhang Y. (2018). Manipulating the degradation behavior and biocompatibility of Mg alloy through a two-step treatment combining sliding friction treatment and micro-arc oxidation. J. Mater. Chem. B.

[48] Yang J., Gu Y., Zhou X., Zhang Y. (2018). Tribocorrosion behavior and mechanism of micro-arc oxidation Ca/P coating on nanocrystallized magnesium alloys. Mater. Corros..

[49] Ballam L.R., Arab H., Bestetti M., Franz S., Masi G., Sola R., Martini C. (2021). Improving the corrosion resistance of wrought ZM21 magnesium alloys by plasma electrolytic oxidation and powder coating. Materials.

[50] Kim K., Yu M., Zong X., Fang D., Seo Y.-S., Hsiao B.S., Chu B., Hadjiargyrou M. (2003). Control of degradation rate and hydrophilicity in electrospun non-woven poly(d,l-lactide) nanofiber scaffolds for biomedical applications. Biomaterials.

[51] Soria J.M., Ramos C.M., Bahamonde O., Cruz D.M.G., Sánchez M.S., Esparza M.A.G., Casas C., Guzmán M., Navarro X., Ribelles J.L.G. (2007). Influence of the substrate’s hydrophilicity on the in vitro Schwann cells viability. J. Biomed. Mater. Res..

[52] Talha M., Ma Y., Xu M., Wang Q., Lin Y., Kong X. (2020). Recent advancements in corrosion protection of magnesium alloys by silane-based sol–gel coatings. Ind. Eng. Chem. Res..

[53] Qiu Z., Yin B., Wang J., Sun J., Tong Y., Li L., Wang R. (2021). Theoretical and experimental studies of sol–gel electrodeposition on magnesium alloy. Surf. Interface Anal..

[54] Nezamdoust S., Seifzadeh D., Habibi-Yangjeh A. (2020). Nanodiamond incorporated sol-gel coating for corrosion protection of magnesium alloy. Trans. Nonferrous Met. Soc. China.

[55] Amaravathy P., Sowndarya S., Sathyanarayanan S., Rajendran N. (2014). Novel sol gel coating of Nb_2_O_5_ on magnesium alloy for biomedical applications. Surf. Coat. Technol..

[56] Ashassi-Sorkhabi H., Moradi-Alavian S., Kazempour A. (2019). Salt-nanoparticle systems incorporated into sol-gel coatings for corrosion protection of AZ91 magnesium alloy. Prog. Org. Coat..

[57] Khramov A.N., Balbyshev V.N., Kasten L.S., Mantz R.A. (2006). Sol–gel coatings with phosphonate functionalities for surface modification of magnesium alloys. Thin Solid Films.

[58] Nezamdoust S., Seifzadeh D., Rajabalizadeh Z. (2019). Application of novel sol–gel composites on magnesium alloy. J. Magnes. Alloy..

[59] Pezzato L., Rigon M., Martucci A., Brunelli K., Dabalà M. (2019). Plasma Electrolytic Oxidation (PEO) as pre-treatment for sol-gel coating on aluminum and magnesium alloys. Surf. Coat. Technol..

[60] Zhang L., Wu Y., Zeng T., Wei Y., Zhang G., Liang J., Cao B. (2021). Preparation and characterization of a sol–gel ahec pore-sealing film prepared on micro arc oxidized AZ31 magnesium alloy. Metals.

[61] Liu Y., Cheng X., Wang X., Sun Q., Wang C., Di P., Lin Y. (2021). Micro-arc oxidation-assisted sol-gel preparation of calcium metaphosphate coatings on magnesium alloys for bone repair. Mater. Sci. Eng. C.

[62] Weng W., Wu W., Yu X., Sun M., Lin Z., Ibrahim M., Yang H. (2020). Effect of gel MA hydrogel coatings on corrosion resistance and biocompatibility of MAO-coated Mg alloys. Materials.

[63] Akram M., Arshad N., Aktan M.K., Braem A. (2020). Alternating current electrophoretic deposition of chitosan–gelatin–bioactive glass on Mg–Si–Sr alloy for corrosion protection. ACS Appl. Bio Mater..

[64] Maqsood M.F., Raza M.A., Ghauri F.A., Rehman Z.U., Ilyas M.T. (2020). Corrosion study of graphene oxide coatings on AZ31B magnesium alloy. J. Coat. Technol. Res..

[65] Antonioc I., Miculescu F., Cotrut C. (2020). Controlling the degradation rate of biodegradable Mg–Zn-Mn alloys for orthopedic applications by electrophoretic deposition of hydroxyapatite coating. Materials.

[66] Wang Z.X., Xu L., Zhang J.W., Ye F., Lv W.G., Xu C., Yang J. (2020). Preparation and degradation behavior of composite bio-coating on ZK60 magnesium alloy using combined micro-arc oxidation and electrophoresis deposition. Front. Mater..

[67] Kang Z.X., Zhang J.Y., Niu L. (2018). A one-step hydrothermal process to fabricate superhydrophobic hydroxyapatite coatings and determination of their properties. Surf. Coat. Technol..

[68] Zhou Z., Zheng B., Gu Y., Shen C., Wen J., Meng Z., Qin A. (2020). New approach for improving anticorrosion and biocompatibility of magnesium alloys via polydopamine intermediate layer-induced hydroxyapatite coating. Surf. Interfaces.

[69] Peng M., Hu F., Du M., Mai B., Zheng S., Liu P., Chen Y. (2020). Hydrothermal growth of hydroxyapatite and ZnO bilayered nanoarrays on magnesium alloy surface with antibacterial activities. Front. Mater. Sci..

[70] Yang Q., Tabish M., Wang J., Zhao J. (2022). Enhanced corrosion resistance of layered double hydroxide films on Mg alloy: The key role of cationic surfactant. Materials.

[71] Yuan J., Yuan R., Wang J., Li Q., Xing X., Liu X., Hu W. (2018). Fabrication and corrosion resistance of phosphate/ZnO multilayer protective coating on magnesium alloy. Surf. Coat. Technol..

[72] Zhu J., Jia H., Liao K., Li X. (2021). Improvement on corrosion resistance of micro-arc oxidized AZ91D magnesium alloy by a pore-sealing coating. J. Alloys Compd..

[73] Liu W., Yan Z., Ma X., Geng T., Wu H., Li Z. (2018). Mg-MOF-74/MgF_2_ composite coating for improving the properties of magnesium alloy implants: Hydrophilicity and corrosion resistance. Materials.

[74] Sun J., Cai S., Wei J., Ling R., Liu J., Xu G. (2020). Long-term corrosion resistance and fast mineralization behavior of micro-nano hydroxyapatite coated magnesium alloy In Vitro. Ceram. Int..

[75] Berndt C.C., Hasan F., Tietz U., Schmitz K.P. (2014). A review of hydroxyapatite coatings manufactured by thermal spray. Adv. Calcium Phosphate Biomater..

[76] Bansal P., Singh G., Sidhu H.S. (2020). Investigation of surface properties and corrosion behavior of plasma sprayed HA/ZnO coatings prepared on AZ31 Mg alloy. Surf. Coat. Technol..

[77] Mardali M., SalimiJazi H.R., Karimzadeh F., Luthringer B., Blawert C., Labbaf S. (2019). Comparative study on microstructure and corrosion behavior of nanostructured hydroxyapatite coatings deposited by high velocity oxygen fuel and flame spraying on AZ61 magnesium based substrates. Appl. Surf. Sci..

[78] Yao H.L., Hu X.Z., Wang H.T., Chen Q.Y., Bai X.B., Zhang M.X., Ji G.C. (2019). Microstructure and corrosion behavior of thermal-sprayed hydroxyapatite/magnesium composite coating on the surface of AZ91D magnesium alloy. J. Therm. Spray Technol..

[79] Daroonparvar M., Khan M.F., Saadeh Y., Kay C.M., Gupta R.K., Kasar A.K., Bakhsheshi-Rad H.R. (2020). Enhanced corrosion resistance and surface bioactivity of AZ31B Mg alloy by high pressure cold sprayed monolayer Ti and bilayer Ta/Ti coatings in simulated body fluid. Mater. Chem. Phys..

[80] Wang Q., Sun Q., Zhang M.X., Niu W.J., Tang C.B., Wang K.S., Wang L. (2018). The influence of cold and detonation thermal spraying processes on the microstructure and properties of Al-based composite coatings on Mg alloy. Surf. Coat. Technol..

[81] García-Rodríguez S., López A.J., Bonache V., Torres B., Rams J. (2020). Fabrication, Wear, and Corrosion Resistance of HVOF Sprayed WC-12Co on ZE41 Magnesium Alloy. Coatings.

[82] Gao Y.L., Liu Y., Song X.Y. (2018). Plasma-sprayed hydroxyapatite coating for improved corrosion resistance and bioactivity of magnesium alloy. J. Therm. Spray Technol..

[83] Yao H.L., Yi Z.H., Yao C., Zhang M.X., Wang H.T., Li S.B., Ji G.C. (2020). Improved corrosion resistance of AZ91D magnesium alloy coated by novel cold-sprayed Zn-HA/Zn double-layer coatings. Ceram. Int..

[84] Singh B., Singh G., Sidhu B.S. (2019). Analysis of corrosion behaviour and surface properties of plasma-sprayed composite coating of hydroxyapatite–tantalum on biodegradable Mg alloy ZK60. J. Compos. Mater..

[85] Mohajernia S., Pour-Ali S., Hejazi S., Saremi M., Kiani-Rashid A.R. (2018). Hydroxyapatite coating containing multi-walled carbon nanotubes on AZ31 magnesium: Mechanical-electrochemical degradation in a physiological environment. Ceram. Int..

